# Tracking Glucose Trends, Unveiling Clinical Patterns: Insights From Continuous Glucose Monitoring in Patients at the Extreme of BMI and Eating Disorders Psychopathology

**DOI:** 10.1002/erv.70057

**Published:** 2025-11-17

**Authors:** Marianna Rania, Concetta Irace, Raffaella Sacco, Mariateresa Bevacqua, Elvira Anna Carbone, Antonio Cutruzzolà, Maria Chiara Pelle, Franco Arturi, Cristina Segura‐Garcia

**Affiliations:** ^1^ Renato Dulbecco University Hospital Catanzaro Italy; ^2^ Department of Health Sciences University Magna Græcia Catanzaro Italy; ^3^ Department of Clinical and Experimental Medicine University Magna Græcia Catanzaro Italy; ^4^ Department of Medical and Surgical Sciences University Magna Græcia Catanzaro Italy

**Keywords:** AN, anorexia, BED, binge‐eating, CGM, eating disorders, glucose, hypoglycemia

## Abstract

**Objective:**

Disturbances of glucose homoeostasis are claimed to act as both a consequence and maintaining factor in eating disorders (EDs). This study explored glucose trends and their association with real‐time food intake and self‐report eating psychopathology in a sample of patients with anorexia nervosa (AN) and binge‐eating disorder (BED).

**Method:**

30 patients (AN, 15; BED, 15) wore continuous glucose monitoring (CGM) sensors while synchronously collecting data on daily food intake. CGM outputs were extracted and correlated with nutritional intake, daily meals composition and self‐report eating psychopathology (BES, NEQ, GQ, Y‐FAS).

**Results:**

Up to 74% of participants experienced hypoglycaemia in the study period, with unique trends by diagnosis (prolonged, interprandial, nocturnal episodes in AN; brief, postprandial, daytime episodes in BED). Significant association between the average number of daily meals, glucose coefficient of variation, and symptomatic events was evident in AN. Self‐report night eating and food addiction symptoms in AN, and self‐report grazing in BED, associated, respectively, with daytime and symptomatic hypoglycaemia.

**Conclusions:**

Hypoglycaemia is a frequent finding in patients with AN and BED and is associated with daily meals composition and dysfunctional eating behaviours. Theoretical explanations are provided for a diagnosis‐specific effect on hypoglycemia events. CGM could valuably contribute to understanding, clinical staging, and customised treatments of EDs.

## Highlights

- Glucose homoeostasis disturbances may stem from and maintain eating disorders.
- Continuous glucose monitoring (CGM) reveals hypoglycaemia is a frequent finding, with distinct patterns—nocturnal in anorexia nervosa, postprandial in binge‐eating disorder.
- Low glucoses associate with pathological eating behaviours (night eating, food addiction, and grazing), suggesting CGM‐based biofeedback could guide personalised interventions.

## 1 Introduction

Eating disorders (EDs) are considered among the most disabling and fatal psychiatric disorders. According to the most updated evidence, people suffering from EDs exhibit a 3.32 higher mortality ratio compared to non‐affected peers (Larsen et al. 2024). This estimate is primarily explained by death from suicide, being EDs associated with several other psychiatric disorders (Udo and Grilo 2019), but a detrimental effect can be hypothesised for health‐related correlates.

EDs are burdened by several short‐ and long‐term medical complications involving several systems and physiological pathways whose altered functions might range from minimally to critically life‐threatening(Yu and Muehleman 2023) Interestingly, early disturbances in glucose homoeostasis have been claimed to either follow (Juarascio et al. 2022) or reinforce the disordered eating behaviours by impairing momentary blood glucose levels and other indirect glucose regulation pathways (Ilyas et al. 2019; Presseller et al. 2020) Further, the genomics applied to psychiatric disorders have recently suggested that EDs share genetic variations in loci implicated in the regulation of glucose metabolism and metabolic diseases such as type 2 diabetes and obesity, hypothesising that individuals suffering from EDs might be vulnerable to metabolic dysfunctions even before the onset or the effects of the disorder itself (Hudson et al. 2020).

Exploring glucose abnormalities in the early stages of EDs could advance our understanding of the diseases, enable disease staging based on the severity of the metabolic impairment, and enable tailored nutritional and pharmacological interventions.

Very recently, the application of continuous glucose monitoring technology (CGM) to EDs has been suggested for its compelling potential in evaluating intra‐day and inter‐day glucose variability(Presseller et al. 2020). CGM devices, so far approved and used for diabetes, have revolutionised the capacity to measure interstitial glucose levels, interpret glucose data, and adjust therapy accordingly in the daily‐life environment. Up to date, five studies have applied CGM technology to EDs (Germain et al. 2023; Juarascio et al. 2022; Presseller et al. 2022, 2024; Uotani et al. 2022) and some other used similar approach to adolescents with obesity (Kishimoto and Ohashi 2022, 2023; Naguib et al. 2022; Vidmar et al. 2021).

In the study of Germain and colleagues, 28 women with Anorexia Nervosa (AN) underwent CGM over 5 days and psychometric, metabolic, and nutritional features were recorded (Germain et al. 2023). Notably, the proportion of time spent below the range (TBR) spanned from 21% to 52%, markedly exceeding the ∼3% typically reported in normoglycemic individuals (Tokutsu et al. 2020). Nutritional intake, available for a subset of patients, revealed a discriminant association between carbohydrate intake and pre‐ and post‐breakfast, and pre‐ and post‐dinner glucose data.

Uotani and colleagues estimated glucose variation, hypoglycaemia, and hyperglycaemia in 18 patients with AN and Bulimia Nervosa (BN) by performing a 5‐day‐long CGM (Uotani et al. 2022). Higher glucose variability, expressed as mean amplitude of glycaemic excursion, and hypoglycaemic events were significantly more frequent in patients with binge‐eating, whose episodes were mostly nocturnal with respect to AN.

Two more studies applied CGM to the binge‐eating spectrum (Juarascio et al. 2022; Presseller et al. 2022). In the first study, a 12‐week long trial with CGM and a just‐in‐time adaptive intervention system based on cognitive behavioural therapy (CBT) was tested on 30 participants within the binge‐eating spectrum (BN; Binge‐Eating Disorder, BED) (Juarascio et al. 2022). Results suggested that delivering momentary interventions based on CGM analysis (ON mode) was able to influence eating psychopathology cognitions and behaviours. In the second study, 52 individuals with binge‐eating (BN, BED) completed 1 week‐long CGM together with ecological assessment of eating behaviours (Presseller et al. 2022). Compared to the healthy control group (*N* = 22), a straightforward association emerged between glucose variability, defined as the standard deviation of the CGM data, across the 7‐day study period and binge‐eating frequency, but not with interprandial glucose. The latest study of Presseller, and the most recent of all the literature on this topic, employed CGM outputs to train a machine learning algorithm in the ability to detect meal consumption and fasting periods in individuals with BED (Presseller et al. 2024).

More robust and detailed evaluations of CGM‐derived trends matched with momentary nutritional intake and eating behaviours could advance the understanding of the bidirectional relation between disordered eating behaviours and nutritional intake under the mediation effect of glucose variability. Within the objective and CGM monitoring, more targeted interventions and better short‐ and long‐term outcomes could be expected.

The present study investigated CGM metrics in a sample of individuals suffering from AN and BED. It further explored the association between relevant CGM outputs, real‐time food intake, daily meals composition, and self‐report eating psychopathology.

## 2 Methods

This cross‐sectional study incorporated both descriptive and analytical approaches. Descriptive analyses served to explore CGM‐derived metrics in individuals suffering from AN and BED (aim 1). Analytical techniques were used to exploratory delve into the relation between CGM outputs and the ecological momentary assessment reporting food intake, daily meals composition and eating behaviours during the study period (aim 2).

The protocol was submitted and approved by the Local Ethical Committee Regione Calabria, Area Centro (n. 128, April 16^th^, 2020; n. 65, February 18^th^, 2021).

### 2.1 Participants

Participants were recruited among those seeking treatment for EDs to the Outpatient Unit for Clinical Research and Treatment of Eating Disorders embedded within the Psychiatric Unit of the University Hospital Renato Dulbecco, Catanzaro, Italy (May 2020‐July 2022). Diagnoses of EDs were performed by psychiatrists through the clinician‐administered Eating Disorder Examination 17.0 interview (Calugi et al. 2015). Demographic and anamnestic information were then collected for eligibility. Inclusion criteria were age 16–60 years, diagnosis of AN (restrictive) or BED, willingness to participate, ability to answer self‐report questionnaires, and valid informed consent. Individuals suffering from major psychiatric disorders other than EDs (e.g., schizophrenia or other psychotic disorders, bipolar disorder, substance use disorders), medical diseases or medications able to affect cognitive functions or glycaemic homoeostasis (e.g., diagnosed diabetes, metabolic syndrome), and being pregnant, having recently given birth or breastfeeding were excluded. Eligibility also included no ongoing psychiatric medications, nutritional therapy, or active exercise of any type. Eligible participants were duly informed about the aims of the study and the procedures and were informed that participating was voluntary and free from any compensation. A valid informed consent was obtained before any study procedure took place (for participants < 18 years old, parents or legal tutors were asked to sign the informed consent).

Considering the study was designed to reveal trends and generate hypotheses for future research, an a priori computation for medium‐to‐large effect sizes was conducted (Gpower 3.1). Out of the minimum of 29 participants sufficient for detecting a correlation of ρ ≥ 0.45 (two‐tailed, *α* = 0.05, power = 0.80), 35 participants (AN, 19; BED, 16) were considered eligible and recruited.

### 2.2 Procedures

After screening for eligibility, further anamnestic data were retrieved (e.g., maximum weight loss after a diet, duration of untreated illness) and psychometric questionnaires were administered to participants to assess eating psychopathology and eating behaviours severity.

An experienced dietician performed the anthropometrical evaluation (i.e., body mass index, kg/m^2^; BMI), and instructed participants about the food diary compilation. Finally, the sensor was inserted by the participant's referred physician within the clinic on the back/side region of the upper arm or abdomen according to the participant preference. All the details regarding the system's disposable sensor and the handheld device were provided. Participants wore the device for a minimum of 10 days to a maximum of 2 weeks period. The sensor was then removed, and CGM outputs downloaded through specific platforms. Food diaries were collected for the analyses.

#### 2.2.1 Psychometric Assessment

The follow self‐report questionnaires were administered:

- The Binge Eating Scale (BES) (Di Bernardo et al. 1998; Gormally et al. 1982; Ricca et al. 2000) a 16‐items questionnaire measuring cognitions, feelings, and behaviours associated with binge eating. In this study, total BES score was used as a measure of binge‐eating psychopathology severity.
- The Grazing Questionnaire (GQ) (Aloi, Rania, De Fazio, et al. 2017; Lane and Szabó 2013), consisting of eight items assessing grazing behaviours, controllability, and the overall grazing severity (higher scores, higher severity). In the current study all dimensions were used for the analysis.
- The Night Eating Questionnaire (NEQ) (K. C. Allison et al. 2008; Aloi, Rania, De Fazio, et al. 2017), that assesses four dimensions of night eating (morning anorexia, evening hyperphagia, mood/sleep, nocturnal ingestions) by 14 items. In the current study, all subdimensions were used for the analysis.
- The Yale Food Addiction Scale 2.0 (Y‐FAS 2.0) (Aloi, Rania, Rodríguez Muñoz, et al. 2017; Gearhardt et al. 2016), a 35‐items questionnaire assessing addiction‐like eating behaviours across 11 criteria over the past 12 months. The sum of endorsed criteria yields a total score (0–11), positivity to the scale (2–3 symptoms plus the impairment dimension), and a severity level (mild: two to three symptoms, moderate: 4–5 symptoms, severe > 6 symptoms) were used for the analysis.

#### 2.2.2 Food Intake Assessment

Participants were asked to track and self‐report all eating episodes, either main meals (i.e., breakfast, morning snack, lunch, afternoon snack, dinner) or other episodes (e.g., more snacking, binge‐eating, night eating) whenever they eat, over the study period (time spent wearing the device). They were instructed to report whether the meals were not completed and to report on the diary, whenever within the trial, the presence of disabling symptoms such as hand tremor, sweating, palpitations, impaired vision, and fainting suggestive of hypoglycaemia. Food diaries were reviewed by the dietician, who profiled the recorded items according to food groups categories (e.g., cereals, fish/meat/eggs, vegetables, fruits).

The number of events (i.e., number of meals, skipping meals, breakfast, morning and afternoon snacks, lunch, dinner, night eating) or food groups portions (e.g., cereals, vegetables, fruits) consumed over the study period were extracted and means per day were computed (number of events/days wearing the device). The ‘hours between meals’ feature was computed as the sum of fasting hours between meals/number of meals.

#### 2.2.3 CGM

Real‐time CGM (rtCGM) was run with Dexcom G6 (Dexcom Inc., California, USA) in individuals with BED (Acciaroli et al. 2023) and intermittently scanned CGM (isCGM) with FreeStyle Librae (Abbott Laboratories, Illinois, USA) in individuals with AN (Blum 2018). This choice was clinician guided. De facto, given its factory technology, FreeStyle Librae allows for the complete management of the receiver to be entrusted to the primary caregiver. isCGM, also known as ‘flash’ glucose monitoring, measures glucose levels continuously but requires on‐demand sensor scanning for the visualisation and storage of glucose values. For an accurate and complete recording, the caregivers of participants with AN were instructed to scan the sensor at least every 8 hours—the maximum data storage interval—as well as in the event of symptoms suggestive of high or low glucose levels. Threshold alarms were not set for either of the CGM systems. The urgent low soon alarm on the Dexcom G6, which predicts impending glucose level < 55 mg/dL, cannot be disabled. Recruitment was consecutive for the two diagnostic groups.

The values recorded by the CGM were grouped into Time in Range (TIR: 70–180 mg/dL), Time Below Range (TBR: < 70 mg/dL), and Time Above Range (TAR: > 180 mg/dL), in accordance with an international consensus (Battelino et al. 2019). CGM data collected for the current study were mean plasma glucose (mg/dL), glucose management indicator (GMI, %), coefficients of variation (CV, %), TIR (%), TAR (%), TBR (%), and time below 54 mg/dL (clinically significant hypoglycaemia). The GMI is an estimate of laboratory‐measured glycated haemoglobin based on CGM mean glucose level. Rough data were also computed to extract further features for hypoglycaemic episodes. A hypoglycaemic episode was defined as any episode characterised by a glucose value < 70 mg/dL and lasting ≥ 15 min. For the analysis, hypoglycaemic events were also recoded based on the symptoms (i.e., self‐reported in the ecological momentary assessment survey), timing (daytime, nighttime; average of episodes, %), and type (postprandial, interprandial; average of episodes, %). More specifically and according to the ambulatory glucose profile (AGP), the visual report that summarises the CGM glucose data according to day hours, nighttime hypoglycaemia was considered if occurring between 00h00 and 06h00 a.m., and daytime if occurring between 06h00 and 00h00. Daytime hypoglycaemia was further classified as postprandial (events recorded in the 4 h after food intake) (Altuntaş 2019), and interprandial (episodes occurring 4 h after the prior meal or between 06h00 a.m. and the following meal).

### 2.3 Statistical Analysis

The analysis was performed using JASP software, version 0.17.1 (Apple Silicon). All CGM outputs were standardised to account for differences in sensor wear duration (10 days for Dexcom G6, 14 days for FreeStyle Librae). Shapiro‐Wilk normality test was applied to investigate the normal distribution of the features (supplementary material, Supporting Information S1: Table S1–S2). Descriptive analysis was run to evaluate CGM, food diary composition and daily meals composition, and self‐report eating psychopathology outputs for the two groups. Data are shown as means and standard deviations, percentages and standard deviations, frequencies and percentages, as appropriate. Spearman's rank correlation test served to investigate associations between relevant anthropometric and anamnestic variables, glycaemic outputs, daily number of meals and food diary contribution and self‐report eating psychopathology. Specifically, three correlation analyses were run: a) CGM outputs, BMI, age, DUI, self‐report measures from BES, NEQ, GQ, Y‐FAS 2.0; b) CGM outputs and meals composition extracted from food diary (e.g., average meals per day, breakfasts); c) CGM outputs, daily nutritional contribution (e.g., cereals per day, vegetables per day). The three analyses were run, separately, in the two groups. To enable a balanced interpretation of results, a dual‐threshold approach was adopted to acknowledge both the Type I error control and the hypothesis‐generating aim of the study. Bonferroni correction was applied within conceptually defined families of tests (each CGM output defined as an independent family), resulting in a strict corrected threshold of *α* = 0.005 (*α* = 0.05/10 variables tested). On the other hand, correlations yielding *p*‐values between 0.005 and 0.025 were interpreted as relevant trends deserving clinical attention and further investigation in larger confirmatory studies (all significant results before correction in the supplementary material for full transparency).

## 3 Results

One participant with BED dropped the procedure due to an inflammatory skin reaction at the site of the sensor application. Thus, the overall tolerance was optimal. Three more participants with AN were excluded from the analyses for incomplete food diary compilation (*N* = 2) and nutritional support need (*N* = 1). The final sample completing the trial was 30 (AN, *N* = 15; BED, *N* = 15). All participants were female and white; the mean age was 17.2 ± 4.1 for AN and 40.2 ± 1.0 for BED. Table 1 shows anthropometric, anamnestic, and CGM data for both groups.

**TABLE 1:** Anthropometric, anamnestic, and CGM data in participants with AN and BED.

	AN		BED	
	Mean	SD	Mean	SD
BMI (kg/m^2^)	17.1	2.0	40.9	6.1
Max weight loss (kg)	12.3	4.9	24.6	10.1
DUI (months)	19.2	17.5	268.3	139.3
Mean plasma glucose (mg/dL)	88.3	8.6	113.1	10.5
GMI (%)^a^	5.4	0.2	5.8	0.4
CV (%)^a^	14.7	2.7	16.2	3.4
TIR (%)^a^	92.6	10.1	98.1	2.8
TAR (%)^a^	0.1	0.3	0.8	1
TBR (%)^a^	7.3	10.0	1.1	2.7
Time below 54 mg/dL (%)^a^	0.01	0.3	0.2	0.5

*Note:* Data are expressed as means and standard deviation (SD). Data are available for all the sample (AN, 15; BED, 15).

Abbreviations: BMI, body mass index; DUI, duration of untreated illness (months); CV, coefficient of variation; GMI, glucose management indicator; TAR, time above the range; TBR, time below the range; TIR, time in range.

^a^ Means of percentages and standard deviations.

Time spent in the target range was very high in both groups (> 90%), with a TBR of 1.1% and 7.3% in participants with BED and AN, respectively.

Hypoglycaemia events during the selected AGP period are described in Table 2.

**TABLE 2:** Hypoglycaemia events in the study period.

	AN		BED	
	Mean	SD	Mean	SD
Participants experiencing at least one episode^b^	12	80	10	66.7
Mean episodes (mean/day)	0.9	0.9	0.3	0.5
Mean value (mg/dL)	63.8	4.4	58.2	7.6
Mean duration (minutes)	149.7	79.6	36.5	34.7
Participants experiencing symptoms^b^	2	16.7	3	30
Mean symptomatic events (mean/day)	0.1	0.4	0.1	0.1
Interprandial hypoglycemia^a^	78.2	20.4	26.8	44.1
Interprandial hypoglycemia episodes (mean/day)	0.6	0.5	0.1	0.3
Postprandial hypoglycemia^a^	21.8	20.4	73.2	44.1
Postprandial hypoglycemia episodes (mean/day)	0.3	0.4	0.2	0.2
Daytime hypoglycemia^a^	31.7	29.2	86.9	32.1
Daytime hypoglycemia episodes (mean/day)	0.5	0.6	0.3	0.3
Nighttime hypoglycemia^a^	68.3	29.2	13.1	321
Nighttime hypoglycemia episodes (mean/day)	0.4	0.4	0.1	0.2

*Note:* Data are expressed as means and standard deviation (SD). Postprandial hypoglycaemia: events recorded in the 4 h after food intake; interprandial hypoglycaemia: episodes occurring 4 h after the prior meal or between 06h00 a.m. and the following meal; nighttime hypoglycaemia: events occurring between 00h00 and 06h00 a.m.; daytime hypoglycaemia: events occurring between 06h00 a.m. and 00h00. Data are available for all the sample (AN, 15; BED, 15).

^a^ Means of percentages and standard deviations.

^b^ frequencies and percentages.

Up to 74% of participants experienced at least one hypoglycaemic event (80% AN, *N* = 12; 66.7% BED, *N* = 10), with distinct mean durations by diagnosis (149.7 ± 79.6 min in AN; 36.5 ± 34.7 min in BED). The mean glucose value during hypoglycaemia was 63.8 ± 4.4 mg/dL in AN and 58.2 ± 7.6 mg/dL in BED. Hypoglycaemia was asymptomatic in the 83% of participants with AN and in 12 out of 15 with BED (70%), with symptoms reported only for daytime events. By type and time, hypoglycaemia was more frequently endorsed in the interprandial state (78.2%) and at night in AN (68.3), whilst BED mostly suffered from daytime (86.9%) and postprandial episodes (73.2%) (Table 2).

Results from the psychometric questionnaires are shown in Supporting Information S1: Table S3. Food diary composition and derived features by groups in Supporting Information S1: Table S4.

Exploratory analysis in AN revealed a significant association between the average number of daily meals (i.e., mean breakfasts and dinners, main meal skipping), CV, and symptomatic events (Table 3). Specifically, variations in glucose levels (CV) were inversely associated with skipping main meals (*ρ* = −0.608; *p* = 0.016) and the number of dinners consumed (*ρ* = −0.661; *p* = 0.019), and positively associated with consuming more breakfasts (*ρ* = 0.695; *p* = 0.004); on the other hand, the lower the number of breakfasts and dinners consumed in the study period, the higher the number of symptomatic events per day (*ρ* = −0.666; *p* = 0.018; *ρ* = −0.813; *p* = 0.001, respectively). Further, significant associations emerged between self‐report night eating and positivity to food addiction (FA) reported by participants with AN with some glucose derived measure such as CV and daytime hypoglycaemia. Notably, lower scores on night eating examination were correlated to higher CV (*ρ* = −0.762; *p* = 0.002; results for NEQ total score), whereas positivity to FA was correlated to higher CV (*ρ* = 0.662; *p* = 0.019) and lower episodes of daytime hypoglycemia per day (*ρ* = −0.718; *p* = 0.009) (results without Bonferroni correction in Supporting Information S1: Table S5).

**TABLE 3:** Spearman's correlation between CGM outputs, food diary and self‐report pathological eating behaviours.

		*ρ*	*p*
Anorexia nervosa			
CV	*main meal skipping*	−0.608	0.016
	*breakfast*	0.695	0.004
	NEQ mood sleep	−0.744	0.002
	NEQ nocturnal ingestion	−0.758	0.002
	NEQ total	−0.762	0.002
	*dinner*	−0.661	0.019
	Y‐FAS 2.0 positivity	0.662	0.019
Daytime hypoglycemia	Y‐FAS 2.0 positivity	−0.718	0.009
Symptomatic hypoglycemia	*breakfast*	−0.666	0.018
	*dinner*	−0.813	0.001
Binge eating disorder			
TAR	*cereals*	0.609	0.016
	*sweet snacks*	0.639	0.010
Daytime hypoglycemia	GQ grazing behaviours	−0.661	0.019
Symptomatic hypoglycemia	GQ total	0.731	0.025

*Note:* Italics for mean portions/main meal per day calculated from food diaries. Dual‐threshold interval for clinical relevance set at 0.005–0.25; Strict Bonferroni corrected significant levels set at *p* < 0.005 (for results w/o correction, refer to Supporting Information S1: Tables S5–S6). Daytime hypoglycaemia: events occurring between 06h00 a.m. and 00h00. Data are available for all the sample (AN, 15; BED, 15).

Abbreviations: CV, coefficient of variation; GQ, grazing questionnaire; NEQ, night eating questionnaire; TAR, time above the range; Y‐FAS, yale food addiction scale.

The same analysis conducted on the sample with BED revealed an overall association between self‐report grazing and daytime and symptomatic hypoglycaemia events (Table 3). Specifically, the higher the scores on self‐report grazing the lower the mean episodes of daytime (*ρ* = −0.661; *p* = 0.019) but symptomatic hypoglycemia per day (*ρ* = 0.731; *p* = 0.025). Lastly, a direct association was observed between consumption of cereals and sweet snacks with more time spent above the target range in the same sample (*ρ* = 0.609, *p* = 0.016; *ρ* = 0.639; *p* = 0.010, respectively). After the dual‐threshold correction, several associations in both groups dropped, mostly affecting correlations between CGM outputs and macronutrients composition of food diaries or NEQ subscales (for reference see Supporting Information S1: Table S6).

## 4 Discussion

The present study evaluated CGM‐derived glucose metrics and their association with real‐time food intake, daily meals composition, and self‐report eating psychopathology in individuals suffering from AN and BED.

So far, few studies have explored glucose homoeostasis using modern technologies such as CGM in these clinical populations (Germain et al. 2023; Juarascio et al. 2022; Presseller et al. 2022, 2024; Uotani et al. 2022). This study adds to the field a more detailed characterisation of glucose metrics and new insights on the associations between glycaemia in real‐life and either real‐time food intake and daily meals organisation or self‐report disordered eating behaviours in a sample of AN. It is surely the first in its genre applied to BED, clinically diagnosed, for whom no such evidence has been reported.

Glucose values stayed within the normal range for most of participants (AN, 92%; BED 98%). Despite the overall optimal time in range, noticeable percentages of time spent below 70 mg/dL were evident among individuals with AN. Spending 7% of their time below the range, this population showed twice the recommended target for individuals with normal fasting glucose and normal glucose tolerance (Jarvis et al. 2023; Klonoff et al. 2023).

Particularly striking was the prevalence of individuals experiencing at least one hypoglycaemic episode as well as the high prevalence of asymptomatic hypoglycaemia in either AN or BED. Hypoglycaemia events were endorsed in up to 74% of the sample, above all in participants suffering from AN (80%). Present results are consistent although subtly lower than those reported by Germain (91%), whose sample, though, consisted of patients hospitalised, with lower BMI and a longer duration of illness, making a higher number of hypoglycaemia episodes more likely (Germain et al. 2023). On the contrary, Uotani did find hypoglycaemia events in the binge‐spectrum (AN bp, BN) but not in the restrictive subtype, probably because of the small sample (*N* = 5) and the recording during a lower period (5 days) (Uotani et al. 2022). No such reference, to date, exists for individuals suffering from BED, whose hypoglycaemia percentages using CGM are preliminary new and warrant further investigation.

First in its exploratory nature, this study characterised hypoglycaemia suggesting a diagnosis‐specific effect on the glucose pattern. Unique phenotypes emerged by diagnosis regarding the type, timing, and mean duration of the hypoglycaemia events.

In AN, hypoglycaemia was more frequently interprandial and endorsed at night, with a very long mean duration (more than 2 hours‐long), confirming the findings of Germain's group, but with a threefold higher proportion of nocturnal hypoglycaemia compared to daytime episodes observed in the clinical sample (67% vs. 21%) (Germain et al. 2023). Severe and persistent restriction could explain either the interprandial type, nocturnal presentation or the long duration found in AN. As such, nighttime hypoglycaemia might result from either strict carbohydrates intake during dinner or impaired hepatic glycogen storage responsible for the nocturnal gluconeogenesis (Germain et al. 2023). Further, malnourishment could also interfere with catecholamine production, reliably accounting for an impaired counterregulatory reaction to hypoglycaemia which inevitably lasts longer than it should. Present study lends partial support to these hypotheses. Although cereals intake did not, the number of dinners endorsed in the study period resulted strongly associated with interprandial and longer hypoglycaemia events, beyond correction; daytime hypoglycaemia, although less frequent, was also experienced by participants.

At the other clinical extreme, participants with BED underwent more diurnal, postprandial and shorter hypoglycaemia events. While no similar findings on ecological (real‐life) glucose assessment have been documented so far for BED, recent laboratory‐based evidence has reported postprandial hypoglycaemia in individuals suffering from BED using the extended 5‐h long oral glucose tolerance test (Rania et al. 2023). In this study, a larger cohort of individuals with obesity but without diabetes was examined for hypoglycaemia events according to the presence of binge‐eating, food addiction and the comorbid phenotype. Higher frequencies of reactive hypoglycaemia 2–5 h after the stimulation were evident in the sample with BED or FA, being more strongly associated with food addiction severity than with BMI, glucose phenotype (e.g., impaired fasting glucose) or insulin resistance. The authors hypothesised that this phenomenon could arise from an exaggerated but delayed insulin response to glucose intake, potentially mediated by a combined defect in the first phase of insulin secretion or dysregulated incretin system (Rania et al. 2023). An insulin‐dependent mechanism boosting glucose effectiveness has also been proposed from other authors (Sedgwick and Greenwood 2015). Post‐prandial hypoglycaemia—in the early stages and in the absence of diabetes— has been hypothesised to potentially act as a neurobehavioral reinforcement of the loss of control overeating, suggesting a dynamic interplay between metabolic and behavioural dysregulation (e.g., craving triggered by hypoglycaemia). Present findings complement those reported by Rania and colleagues by capturing postprandial hypoglycemia events in an ecological context which encompass real‐life dietary habits, stressors and behavioural patterns.

Glucose variability, assessed by the coefficient of variation of mean glucose, stayed within the range of 15%–18% assessed for healthy individuals with no diabetes (Shah et al. 2019) for both groups. However, the analysis of association between glucose variability and daily meals composition further suggested that overall meals per day, especially breakfasts, straightforward correlate with CV in AN, such that skipping meals strongly relates with lower glucose variation. If a low coefficient of glucose variation is expected and desirable in type I or II diabetes, as it reflects an optimal pharmacological management of the underlying disease, no data exists on coefficients of variation in individuals experiencing significant weight fluctuations due to AN. It could be hypothesised that the low glycaemic variability observed in this population—likely resulting from strict glucose supplies, impaired gluconeogenesis and blunted counterregulatory reactions to hypoglycemia—may represent a negative prognostic marker for the overall health status and the risk for complications. Consistently, a clear concordance between self‐report pathological eating behaviours and glucose variability was also evident. Lower glucose variability was associated with self‐report night eating, and symptoms of food addiction were highly reported from patients endorsing interprandial and more severe hypoglycaemia. No effect of daily meals composition on glucose variability was evident in BED. Secrecy, feeling of guilt, shame and disgust are core features of the binge‐eating phenomenon, often leading patients to underreport or deny episodes of loss of control overeating (Bremer et al. 2023). CGM technology has the great potential to bio feed clinicians on glycaemic excursions that could be missed in the self‐report food diary and that could relate to pathological eating behaviours (e.g., binge eating) (Presseller et al. 2024). In this specific case, participants were instructed about the information gathered from the device itself and this might have biased participants attitude towards the daily meal organisation or losing control overeating during the experimental phase (no binge‐eating episodes self‐reported). Specifically, greater self‐regulation or inhibition of binge‐eating episodes might have occurred because of the awareness of being monitored—an effect known as the Hawthorne effect (Sedgwick and Greenwood 2015), potentially masking glycaemic excursions associated to binge‐eating episodes. Nevertheless, the specific glycaemic pattern in response to binge eating in real life remains to be elucidated and requires similar CGM‐based monitoring studies in naturalistic settings.

In 2014 Segura‐Garcia and colleagues suggested that daily macronutrient intake could regulate and be regulated by peripheral neuropeptides and that this bidirectional relationship could explain food choice in EDs (Segura‐García et al. 2014). However, only the association between cereals/sweet snacks consumption and higher time spent above the range in BED survived the analysis. Further, no self‐report pathological eating behaviours but grazing showed a significant association with glucose metrics in participants with BED (the association between night eating and interprandial hypoglycaemia dropped after adjusting for multiple comparison). According to some authors, unstructured, unplanned and repetitive eating of small amounts of food after planned meals is subjectively reported by patients suffering from BED instead of proper binge‐eating episodes according to DSM description (S. Allison and Timmerman 2007). Whether the feature ‘loss of control’ was queried before, there is now more support for this specifier to characterise grazing behaviour, contributing to much more similarities than differences with binge‐eating (Kofman et al. 2010; Lane and Szabó 2013; Saunders 2004). Present data corroborate the relevance of grazing behaviour and total score when evaluating daytime and symptomatic hypoglycaemia, respectively. This finding, along with the association between night eating/food addiction and hypoglycaemia in the AN sample, is being reported for the first time.

While far from inferring that self‐reported altered eating behaviours result from the underlying metabolic condition (e.g., night eating and food addiction symptoms as secondary to hypoglycaemia in AN), this remains an area worth further investigation. On one hand, it might hinder significant potential for identifying patients who, experiencing hypoglycaemia, may be at a higher risk of diagnostic cross‐over. On the other, it might inform on the diverse pathophysiology of early metabolic impairment in these disorders and on specific nutritional targets when coupled with real‐time nutritional and eating behaviours assessment.

Strengths of this study include the simultaneous assessment of self‐report eating psychopathology and real‐time food intake and daily meals composition, enabling both a subjective (patient centred) and objective (clinician centres) measure of the same features.

This is, to date, the first investigation of glucose trends in a clinical sample suffering from BED. Present results expand on the previous studies on AN providing data in a sample of younger participants at their first medical contact, with only restrictive features and less severe clinical frame (outpatients, higher BMI, lower duration of illness) (Germain et al. 2023; Uotani et al. 2022). The stringent eligibility criteria with a sample unbiased for medications, nutritional treatments, and exercise of any type should also be acknowledged. Notwithstanding the strengths and potentialities, some limitations should be considered.

First, although the study was adequately powered to detect large effects—consistent with the aim to highlights trends and generate hypotheses for future confirmatory research— the sample size was relatively small. The sample lacked diversity, as participants were all female and white. Accordingly, present findings might not be generalisable to males and other ethnic backgrounds. The inclusion of a healthy control group would have strengthened the methodological robustness of the study design but was not feasible due to practical and regulatory constraints. Participation in studies involving procedures that might be perceived as ‘invasive’ by healthy individuals typically requires financial incentives, which are not permitted under the Italian low. Additionally, CGM devices are not free of charge for individuals not diagnosed with diabetes and no specific fundings were received to support device acquisition for healthy volunteers. Future research including larger and more diverse cohorts, and dedicated fundings to overcome these critical aspects, are warranted for these findings to be replicated and generalisable to the whole population that might be affected. Biases due to recall or uncomplete adherence to self‐report eating behaviours should be considered. Additionally, participants' awareness of being monitored might have introduced a reactivity bias, and the absence of self‐report binge‐eating episode and corresponding glycaemic excursion should be interpreted in the context of this reactivity. In general, CGM systems are recognised to be less accurate in children than in adults, especially for lower glucose values. Moreover, severe dehydration may also contribute to accuracy. Overall, sensor readings in participants with AN could have been less accurate than those examined in participants with BED. Further, two different CGM technologies were used to assess glycaemic trends in the two groups. Although present study did not aim to compare trends between the groups, the use of two different technologies might have contributed to differences in measurement accuracy. Further studies aiming at comparing these populations should address these gaps, possibly evaluate the inference of blind/unblind technology for glucose feedback and define the best device for both populations accordingly.

In conclusion, the burden and the overall risk for death of short‐ and long‐term medical complications is high in EDs, especially for those at the extremes of the body mass index. Some of these medical correlates follow the eating behaviours (e.g., fasting, binge‐eating) but could also reinforce the eating disorder itself. This might be the case for the glucose homoeostasis (Ilyas et al. 2019; Juarascio et al. 2022; Presseller et al. 2020). CGM could help gaining insight on the intricate relation between food consumption, disordered eating behaviours and glycaemic dynamics. This could, accordingly, empower the clinical staging, enable the best treatment choice, tailor the nutritional intervention basing on the specific glycaemic phenotype, cooperate within the usual treatment, and ameliorate the outcomes.

## Author Contributions

**Marianna Rania:** conceptualization, methodology, project administration, supervision, data curation, formal analysis, investigation, writing – original draft, writing – review and editing. **Concetta Irace:** conceptualization, methodology, project administration, supervision, writing – review and editing. **Raffaella Sacco:** investigation, data curation. **Mariateresa Bevacqua:** investigation, data curation. **Elvira Anna Carbone:** investigation, data curation. **Antonio Cutruzzolà:** investigation, data curation. **Maria Chiara Pelle:** investigation, data curation. **Franco Arturi:** conceptualization, methodology, project administration, supervision, writing – review and editing. **Cristina Segura‐Garcia:** conceptualization, methodology, project administration, supervision, formal analysis, writing – review and editing.

## Funding

The authors have nothing to report.

## Conflicts of Interest

C.I. has provided advisory board services for Abbott, Ascensia, Medtronic, Menarini, Novo Nordisk, Roche Diabetes Care Italy, and Senseonics; and has received speaker fees from Abbott, Ascensia, Boehringer Ingelheim, Eli Lilly, and Novo Nordisk

A.C. has received speaker fee from Roche Diabetes Care Italy and research grant from Roche Diabetes Care Italy and NovoNordisk.

## Supporting information


Supporting Information S1

## Data Availability

The data that support the findings of this study are available from the corresponding author, upon reasonable request.
