# Supplementary material for: Tracking Glucose Trends, Unveiling Clinical Patterns: Insights From Continuous Glucose Monitoring in Patients at the Extreme of BMI and Eating Disorders Psychopathology
Source: Eur Eat Disord Rev. 2025 Nov 17;34(3):627–36. doi: 10.1002/erv.70057 (PMC13048741; doi:10.1002/erv.70057)
Supplement: Supplementary file 1 — Supporting Information S1 [file ERV-34-627-s001.docx]

| Table S1. Full descriptive analysis of anamnestic and CGM-derived features by groups | | | | | | | | |
| --- | --- | --- | --- | --- | --- | --- | --- | --- |
|  |  |  |  |  |  |  |  |  |
|  |  | Mean | SD | Median | Shapiro-Wilk | Shapiro-Wilk p-value | Min | Max |
| BMI (kg/m^2^) | an | 17.1 | 2.0 | 16.7 | 0.967 | ns | 13.6 | 20.7 |
|  | bed | 40.9 | 6.1 | 39.8 | 0.956 | ns | 31.9 | 51.3 |
| Max weight loss (kg) | an | 12.3 | 4.9 | 12.5 | 0.922 | ns | 6.5 | 22.5 |
|  | bed | 24.6 | 10.1 | 20 | 0.879 | ns | 10 | 50 |
| DUI (months) | an | 19.1 | 17.5 | 13.1 | 0.705 | 2.819×10^-4^ | 5 | 72 |
|  | bed | 268.3 | 139.3 | 270 | 0.931 | ns | 72 | 468 |
|  |  |  |  |  |  |  |  |  |
| Mean plasma glucose (mg/dl) | an | 88.3 | 8.6 | 87.0 | 0.961 | .71 | 74.0 | 102.0 |
|  | bed | 113.1 | 10.5 | 111.0 | 0.898 | .09 | 93.0 | 132.0 |
| GMI (%) † | an | 5.4 | 0.2 | 4.4 | 0.914 | .16 | 5.1 | 5.7 |
|  | bed | 5.8 | 0.4 | 5.9 | 0.948 | .49 | 4.9 | 6.4 |
| CV (%) † | an | 14.7 | 2.7 | 14.0 | 0.923 | .22 | 11.0 | 20.0 |
|  | bed | 16.2 | 3.4 | 16.0 | 0.924 | .22 | 11.6 | 21.6 |
| TIR (%) † | an | 92.6 | 10.1 | 97.0 | 0.735 | 6.031×10^-4^ | 69.0 | 100.0 |
|  | bed | 98.1 | 2.8 | 99.0 | 0.664 | 1.052×10^-4^ | 89.3 | 100.0 |
| TBR (%) † | an | 7.3 | 10.0 | 3.0 | 0.740 | 6.873×10^-4^ | 0.0 | 31.0 |
|  | bed | 1.1 | 2.7 | 0.1 | 0.452 | 1.486×10^-6^ | 0.0 | 10.7 |
| Time below 54 mg/dl (%) † | an | 0.1 | 0.3 | 0.0 | 0.284 | 9.834×10^-8^ | 0.0 | 1.0 |
|  | bed | 0.2 | 0.5 | 0.0 | 0.413 | 7.525×10^-7^ | 0.0 | 1.5 |
| TAR (%) † | an | 0.1 | 0.3 | 0.0 | 0.284 | 9.834×10^-8^ | 0.0 | 1.0 |
|  | bed | 0.7 | 1.0 | 0.2 | 0.660 | 9.656×10^-5^ | 0.0 | 4.0 |
|  |  |  |  |  |  |  |  |  |
| Hypoglycemia mean value mg/dl) | an | 63.8 | 4.4 | 65.4 | 0.871 | ns | 56.0 | 69.0 |
|  | bed | 58.2 | 7.6 | 60.0 | 0.888 | ns | 41.5 | 66.0 |
| Hypoglycemia mean duration (minutes) | an | 149.7 | 79.6 | 188.0 | 0.860 | ns | 30.0 | 240.0 |
|  | bed | 36.5 | 34.7 | 18.8 | 0.809 | .019 | 5.0 | 110.0 |
| Hypoglycemia number of | an | 0.9 | 0.9 | 0.4 | 0.825 | .018 | 0.1 | 2.6 |
| events | bed | 0.3 | 0.5 | 0.2 | 0.586 | 3.739×10^-5^ | 0.1 | 1.6 |
|  |  |  |  |  |  |  |  |  |
| Interprandial hypoglycemia* | an | 0.6 | 0.5 | 0.4 | 0.887 | .109 | 0.1 | 1.6 |
|  | † | 78.2 | 20.4 | 72.1 | 0.798 | .009 | 50.0 | 100.0 |
|  | bed | 0.1 | 0.3 | 0.0 | 0.45 | 9.549×10^-7^ | 0.0 | 1.1 |
|  | † | 26.8 | 44.1 | 0 | 0.63 | 1.260×10^-4^ | 0.0 | 100.0 |
| Postprandial hypoglycemia* | an | 0.3 | 0.4 | 0.1 | 0.796 | .008 | 0.0 | 1.2 |
|  | † | 21.8 | 20.4 | 27.9 | 0.798 | .009 | 0.0 | 50.0 |
|  | bed | 0.2 | 0.2 | 0.2 | 0.883 | ns | 0.0 | 0.5 |
|  | † | 73.2 | 44.1 | 100.0 | 0.63 | 1.260×10^-4^ | 0.0 | 100.0 |
| Symptomatic hypoglycemia* | an | 0.1 | 0.4 | 0.0 | 0.377 | 2.517×10^-6^ | 0.0 | 1.2 |
|  | † | 4.4 | 13.6 | 0.0 | 0.381 | 2.656×10^-6^ | 0.0 | 47.2 |
|  | bed | 0.1 | 0.1 | 0.0 | 0.622 | 1.001×10^-4^ | 0.0 | 0.4 |
|  | † | 17.5 | 33.4 | 0.0 | 0.623 | 1.042×10^-4^ | 0.0 | 100.0 |
| Daytime hypoglycemia * | an | 0.5 | 0.6 | 0.2 | 0.79 | .007 | 0.0 | 1.9 |
|  | † | 31.7 | 29.2 | 40.5 | 0.791 | .008 | 0.0 | 100 |
|  | bed | 0.3 | 0.3 | 0.2 | 0.743 | .003 | 0.0 | 1.1 |
|  | † | 86.9 | 32.1 | 100.0 | 0.495 | 3.142×10^-6^ | 0.0 | 100.0 |
| Nighttime hypoglycemia* | an | 0.4 | 0.4 | 0.4 | 0.874 | ns | 0.1 | 1.1 |
|  | † | 68.3 | 29.2 | 59.5 | 0.792 | .008 | 0.0 | 100.0 |
|  | bed | 0.1 | 0.1 | 0.0 | 0.464 | 1.381×10^-6^ | 0.0 | 0.5 |
|  | † | 13.1 | 32.1 | 0.0 | 0.495 | 3.142×10^-6^ | 0.0 | 100.0 |
| * Data are expressed as mean episodes per day. † Means of percentages and standard deviations.  Abbreviations: BMI, body mass index; DUI, duration of untreated illness (months); GMI, glucose management indicator; CV, coefficient of variation; TIR, time in range; TAR, time above the range; TBR, time below the range. Postprandial hypoglycaemia: events recorded in the 4 hours after food intake; interprandial hypoglycaemia: episodes occurring 4 hours after the prior meal or between 06h00 am and the following meal; nighttime hypoglycaemia: events occurring between 00h00 and 06h00 am; daytime hypoglycaemia: events occurring between 06h00 am and 00h00. Data are available for all the sample (AN, 15; BED, 15). | | | | | | | | |

| Table S2. Full descriptive analysis of food diary composition and derived features | | | | | | | | |
| --- | --- | --- | --- | --- | --- | --- | --- | --- |
|  |  |  |  |  |  |  |  |  |
|  |  | Mean | SD | Median | Shapiro-Wilk | Shapiro-Wilk p-value | Min | Max |
| Meals | an | 4.1 | 1.1 | 4.4 | 0.9 | ns | 1.9 | 5.8 |
|  | bed | 5.0 | 1.3 | 4.7 | 0.7 | 4.530×10^-4^ | 3.9 | 9.0 |
| Between-meals hours**‡** | an | 3.1 | 1.0 | 2.9 | 0.8 | .001 | 2.3 | 6.0 |
|  | bed | 2.6 | 0.5 | 2.6 | 0.9 | ns | 2.0 | 3.7 |
| Breakfasts | an | 0.8 | 0.3 | 0.9 | 0.8 | .003 | 0.0 | 1.1 |
|  | bed | 0.9 | 0.3 | 1.0 | 0.8 | .004 | 0.1 | 1.3 |
| Morning snacks | an | 0.5 | 0.4 | 0.3 | 0.9 | ns | 0.0 | 1.3 |
|  | bed | 0.7 | 0.4 | 0.7 | 0.9 | ns | 0.1 | 1.7 |
| Lunches | an | 1.0 | 0.1 | 1.0 | 0.8 | .007 | 0.8 | 1.4 |
|  | bed | 1.0 | 0.1 | 1.0 | 0.9 | ns | 0.7 | 1.3 |
| Afternoon snacks | an | 0.7 | 0.3 | 0.7 | 1.0 | ns | 0.1 | 1.2 |
|  | bed | 0.9 | 0.6 | 0.8 | 0.8 | .014 | 0.1 | 2.7 |
| Dinners | an | 1.0 | 0.1 | 0.9 | 0.8 | .003 | 0.8 | 1.3 |
|  | bed | 1.0 | 0.2 | 0.9 | 0.9 | ns | 0.6 | 1.3 |
| Night eating | an | 0.1 | 0.2 | 0.0 | 0.7 | 1.223×10^-4^ | 0.0 | 0.6 |
|  | bed | 0.5 | 0.5 | 0.3 | 0.8 | .003 | 0.0 | 1.8 |
| Main meal skipping | an | 0.2 | 0.3 | 0.1 | 0.6 | 4.634×10^-5^ | 0.0 | 1.1 |
|  | bed | 0.2 | 0.3 | 0.1 | 0.7 | 1.877×10^-4^ | 0.0 | 0.9 |
| Cereals | an | 2.9 | 1.1 | 3.0 | 1.0 | ns | 1.0 | 4.7 |
|  | bed | 4.5 | 1.4 | 4.1 | 0.9 | ns | 2.7 | 6.7 |
| Legumes | an | 0.1 | 0.2 | 0.0 | 0.7 | 1.339×10^-4^ | 0.0 | 0.6 |
|  | bed | 0.1 | 0.1 | 0.1 | 0.8 | .004 | 0.0 | 0.2 |
| Meat | an | 0.8 | 0.5 | 0.8 | 0.9 | .043 | 0.1 | 2.1 |
|  | bed | 1.2 | 0.6 | 1.2 | 0.9 | ns | 0.3 | 2.0 |
| Fish | an | 0.3 | 0.2 | 0.3 | 0.9 | ns | 0.0 | 0.7 |
|  | bed | 0.3 | 0.2 | 0.3 | 0.9 | ns | 0.1 | 0.8 |
| Eggs | an | 0.1 | 0.1 | 0.1 | 0.9 | .035 | 0.0 | 0.4 |
|  | bed | 0.1 | 0.1 | 0.1 | 0.8 | .004 | 0.0 | 0.2 |
| Milk/derivates | an | 1.1 | 0.7 | 1.2 | 0.9 | ns | 0.1 | 2.6 |
|  | bed | 1.4 | 0.8 | 1.4 | 1.0 | ns | 0.0 | 2.8 |
| Vegetables | an | 1.2 | 0.7 | 1.3 | 1.0 | ns | 0.2 | 2.8 |
|  | bed | 0.9 | 0.5 | 0.8 | 0.9 | ns | 0.3 | 1.9 |
| Fruits | an | 1.3 | 1.0 | 1.0 | 0.9 | ns | 0.0 | 3.1 |
|  | bed | 0.8 | 0.8 | 0.7 | 0.8 | .003 | 0.0 | 3.3 |
| Sweet snacks | an | 0.5 | 0.5 | 0.4 | 0.9 | .033 | 0.0 | 1.9 |
|  | bed | 1.8 | 1.0 | 1.7 | 1.0 | ns | 0.3 | 3.4 |
| Salted snacks | an | 0.1 | 0.2 | 0.0 | 0.6 | 1.363×10^-5^ | 0.0 | 0.6 |
|  | bed | 0.3 | 0.2 | 0.3 | 0.9 | ns | 0.0 | 0.7 |
| Sugar drinks | an | 0.1 | 0.3 | 0.0 | 0.5 | 3.331×10^-6^ | 0.0 | 0.9 |
|  | bed | 0.3 | 0.3 | 0.2 | 0.9 | .029 | 0.0 | 1.0 |
| Junk food | an | 0.0 | 0.1 | 0.0 | 0.6 | 3.098×10^-5^ | 0.0 | 0.2 |
|  | bed | 0.4 | 0.4 | 0.3 | 0.9 | .046 | 0.0 | 1.3 |
| Note: Means are intended as mean portions/main meal per day. ‡ Calculated as: (sum of hours between meals/ number of meals)/ device days. Data are available for all the sample (AN, 15; BED, 15). | | | | | | | | |
|  | | | | | | | | |

| Table S3. Results from eating behaviors questionnaires by groups | | | | | |
| --- | --- | --- | --- | --- | --- |
|  |  | AN | | BED | |
|  |  | Mean | SD | Mean | SD |
| BES | Total score | 15.8 | 10.6 | 25.0 | 5.8 |
| GQ | Grazing behavior | 3.5 | 3.8 | 9.4 | 2.8 |
|  | Grazing controllability | 3.6 | 2.8 | 5.0 | 3.1 |
|  | Grazing total score | 7.1 | 6.2 | 14.4 | 5.0 |
| NEQ | Morning anorexia | 2.6 | 1.6 | 2.8 | 1.6 |
|  | Evening hyperphagia | 2.6 | 2.3 | 4.8 | 3.0 |
|  | Mood sleep | 5.5 | 3.1 | 5.1 | 2.6 |
|  | Nocturnal ingestions | 2.3 | 4.1 | 3.6 | 5.1 |
|  | Total score | 13.0 | 7.7 | 16.2 | 6.8 |
| Y-FAS 2.0 | Total n. criteria | 3.2 | 2.8 | 6.1 | 3.4 |
|  | Positivity† | 6 | 40.0 | 13 | 86.7 |
|  | Severity level |  |  |  |  |
|  | mild | 2 | 13.3 | 2 | 13.3 |
|  | moderate | 2 | 13.3 | 4 | 26.7 |
|  | severe | 2 | 13.3 | 7 | 46.7 |
| † Frequencies and percentages of participants satisfying minimum required criteria according to Y-FAS 2.0.  Abbreviations: BES, binge eating scale; GQ, grazing questionnaire; NEQ, night eating questionnaire; Y-FAS, yale food addiction scale. Data are available for all the sample (AN, 15; BED, 15). | | | | | |

| Table S4. Food diary composition and derived features by groups | | | | |
| --- | --- | --- | --- | --- |
|  | AN | | BED | |
|  | Mean | SD | Mean | SD |
| *Daily meals and eating behaviours* |  |  |  |  |
| Meals | 4.1 | 1.1 | 5.0 | 1.2 |
| Between-meals hours**‡** | 3.1 | 0.9 | 2.6 | 0.5 |
| Breakfasts | 0.8 | 0.3 | 0.9 | 0.2 |
| Morning snacks | 0.5 | 0.4 | 0.7 | 0.4 |
| Lunches | 0.9 | 0.1 | 0.9 | 0.1 |
| Afternoon snacks | 0.7 | 0.3 | 0.9 | 0.6 |
| Dinners | 0.9 | 0.1 | 0.9 | 0.6 |
| Night eating | 0.1 | 0.2 | 0.5 | 0.5 |
| Main meal skipping | 0.2 | 0.3 | 0.2 | 0.3 |
|  |  |  |  |  |
| *Daily food diary contribution* |  |  |  |  |
| Cereals | 2.9 | 1.1 | 4.6 | 1.4 |
| Legumes | 0.1 | 0.2 | 0.1 | 0.1 |
| Meat | 0.8 | 0.5 | 1.2 | 0.6 |
| Fish | 0.3 | 0.2 | 0.3 | 0.2 |
| Eggs | 0.1 | 0.1 | 0.1 | 0.1 |
| Milk/derivates | 1.1 | 0.7 | 1.4 | 0.8 |
| Vegetables | 1.2 | 0.7 | 0.9 | 0.5 |
| Fruits | 1.3 | 0.9 | 0.8 | 0.8 |
| Sweet snacks | 0.5 | 0.5 | 1.7 | 0.9 |
| Salted snacks | 0.1 | 0.2 | 0.3 | 0.2 |
| Sugar drinks | 0.1 | 0.2 | 0.3 | 0.3 |
| Junk food | 0.0 | 0.0 | 0.4 | 0.4 |
| Note: Measures are calculated as mean portions/main meal per day. ‡ Calculated as: (sum of hours between meals/ number of meals)/ device days. Data are available for all the sample (AN, 15; BED, 15). | | | | |
|  | | | | |

| Table S5. Spearman’s correlation between CGM outputs, food diary and self-report pathological eating behaviours in AN (significant results before correction) | | | |
| --- | --- | --- | --- |
|  | | | |
|  | | ρ | p |
| Mean plasma glucose | *salted snacks* | 0.518 | .048 |
| CV | *fruits* | 0.521 | .049 |
|  | *meals* | 0.554 | .035 |
|  | *main meal skipping* | -0.608 | .016 |
|  | *breakfast* | 0.695 | .004 |
|  | *dinner* | 0.547 | .035 |
|  | NEQ mood sleep | -0.744 | .002 |
|  | NEQ nocturnal ingestion | -0.758 | .002 |
|  | NEQ total | -0.762 | .002 |
| Interprandial hypoglycemia | *dinner* | -0.661 | .019 |
|  | Y-FAS 2.0 positivity | 0.662 | .019 |
|  | Y-FAS 2.0 severity | 0.577 | .050 |
| Daytime hypoglycemia | *salted snacks* | 0.584 | .046 |
|  | *dinner* | -0.591 | .043 |
|  | Y-FAS 2.0 positivity | 0.584 | .046 |
| Symptomatic hypoglycemia | NEQ mood sleep | 0.587 | .045 |
|  | Y-FAS 2.0 severity | 0.593 | .042 |
| Hypoglycemia mean value | Y-FAS 2.0 positivity | -0.718 | .009 |
| Hypoglycemia mean duration | *milk/derivates* | -0.582 | .047 |
|  | *breakfast* | -0.666 | .018 |
|  | *lunch* | -0.579 | .048 |
|  | *dinner* | -0.813 | .001 |
|  | NEQ mood sleep | 0.596 | .041 |
|  | Y-FAS 2.0 positivity | 0.613 | .034 |
| Note: Italics for mean portions/main meal per day calculated from food diaries.  Dual-threshold interval for clinical relevance set at .005-0.25; Strict Bonferroni corrected significant levels set at p<.005. Abbreviations: CV, coefficient of variation; NEQ, night eating questionnaire; Y-FAS, yale food addiction scale.  Interprandial hypoglycaemia: episodes occurring 4 hours after the prior meal or between 06h00 am and the following meal; daytime hypoglycaemia: events occurring between 06h00 am and 00h00. Data are available for all the sample (AN, 15; BED, 15). | | | |

| Table S6. Spearman’s correlation between CGM outputs, food diary and self-report pathological eating behaviours in BED (significant results before correction) | | | | |
| --- | --- | --- | --- | --- |
|  | | | | |
|  |  |  | ρ | p |
| Mean plasma glucose | GQ grazing controllability | | -0.577 | .031 |
|  | DUI | | 0.567 | .035 |
| TIR | *legumes* | | 0.557 | .031 |
| TBR | *legumes* | | -0.526 | .044 |
| Time below 54 mg/dl | *fruits* | | -0.569 | .027 |
| TAR | *cereals* | | 0.609 | .016 |
|  | *sweet snacks* | | 0.639 | .010 |
| Interprandial hypoglycemia | NEQ morning anorexia | | 0.691 | .039 |
|  | NEQ total | | 0.712 | .031 |
| Daytime hypoglycemia | GQ grazing behaviors | | -0.661 | .019 |
| Symptomatic hypoglycemia | GQ grazing controllability | | 0.730 | .026 |
|  | GQ total | | 0.731 | .025 |
| Note: Italics for mean portions/main meal per day calculated from food diaries.  Dual-threshold interval for clinical relevance set at .005-0.25; Strict Bonferroni corrected significant levels set at p<.005. Abbreviations: DUI, duration of untreated illness (months); TIR, time in range; TBR, time below the range; TAR, time above the range; GQ, grazing questionnaire; NEQ, night eating questionnaire. Interprandial hypoglycaemia: episodes occurring 4 hours after the prior meal or between 06h00 am and the following meal; daytime hypoglycaemia: events occurring between 06h00 am and 00h00. Data are available for all the sample (AN, 15; BED, 15). | | | | |
